# Exposure to green spaces and schizophrenia: a systematic review

**DOI:** 10.1017/S0033291724001533

**Published:** 2024-09

**Authors:** Louise Marcham, Lyn Ellett

**Affiliations:** School of Psychology, University of Southampton, Southampton, UK

**Keywords:** green space, greenspace, psychosis, schizophrenia, systematic review

## Abstract

The mental health benefits of exposure to green spaces are well known. This systematic review summarizes the evidence of green space exposure for people with schizophrenia spectrum disorders (SSDs), focusing on incidence and mental health outcomes, including mental health symptoms and health service use. The study was pre-registered (PROSPERO ID: CRD42023431954), and conducted according to PRISMA guidelines. Seven databases, reference lists, and gray literature sources were searched. Methodological quality was assessed using The Quality Assessment Tool for Quantitative Studies. 126 studies were screened, and 12 studies were eligible for inclusion. Seven studies found that exposure to green space was associated with a reduced risk of schizophrenia (lowest to highest green space exposure: HRs = 0.62–0.37; IRRs = 1.52–1.18), with five studies reporting a dose-response relationship. Of these studies, four examined childhood exposure and the remainder examined adult exposure. Regarding health service use, proximity to green space was not significantly associated with length of hospital admission, though greater green space exposure was associated with reduced hospital admission rates. Three studies found reduced symptoms of anxiety (*d* = −0.70–2.42), depression (*d* = −0.97–1.70) and psychosis (*d* = −0.94) with greater green space exposure. Exposure to green space reduces the risk of schizophrenia, and there is emerging evidence of the potential benefits of green space for reducing symptoms and health service use among people with SSDs. Future research using experimental and longitudinal designs will provide more robust evidence of the benefits of green space for people with SSDs.

There is a growing body of research exploring the relationship between exposure to green space and mental health benefits. In addition, organizations have advocated for the development and protection of green spaces, with the aim of improving population health and wellbeing (Department for Levelling Up, Housing and Communities, 2023; Public Health England; PHE, 2020; World Health Organisation; WHO, 2017). Green spaces can be defined as areas of grass, shrubs, trees, or other vegetation, situated within or adjacent to an urban area (PHE, 2020), and have also been defined by their composition or use, such as nature reserves, parks, forests, and gardens (Taylor & Hochuli, 2017). Exposure to green space generally refers to how often individuals have contact with, or access to, these environments, but can also include single interventions (WHO, 2016). With a growing trend towards urbanization (United Nations, 2018), there is a need to establish the role of green spaces in conferring mental health benefits, to support the continued integration and maintenance of these areas within urban settings (Barton & Rogerson, 2017; Houlden, Weich, Porto de Albuquerque, Jarvis, & Rees, 2018) and to establish their (potential) therapeutic benefits.

Existing reviews have primarily focused on the benefits of green space in terms of common mental health problems and symptomology. Research has shown that exposure to green spaces is associated with a wide range of mental health benefits (Alcock, White, Wheeler, Fleming, & Depledge, 2014; Tran, Sabol, & Mote, 2022; Van den Berg, Maas, Verheij, & Groenewegen, 2010; Wendelboe-Nelson, Kelly, Kennedy, & Cherrie, 2019), including improvements in mood and reduced levels of stress and mental fatigue (Bowler, Buyung-Ali, Knight, & Pullin, 2010; Gascon et al., 2015; Houlden et al., 2018), effects which have been found across the lifespan (Dzhambov, 2018; Fjaestad et al., 2023; McCormick, 2017; Pun, Manjourides, & Suh, 2018). Greater exposure to green spaces has been associated with a reduced risk of developing depression (Brown et al., 2018; Min, Kim, Kim, & Min, 2017; Sarkar, Webster, & Gallacher, 2018) and anxiety disorders (Gascon et al., 2018), and has been found to reduce symptoms related to anxiety and depression, suggesting potential protective effects for improving mental health (Pun et al., 2018). Studies have therefore advocated for the use of green space as an intervention for public mental health (Maas, Verheij, Groenewegen, de Vries, & Spreeuwenberg, 2006; Soga, Evans, Tsuchiya, & Fukano, 2021).

Exposure to green space could be an effective intervention for managing mental health difficulties. Engaging in activities such as gardening, have resulted in overall improved mental wellbeing and reduction in social isolation (Howarth, Brettle, Hardman, & Maden, 2020). For example, accessing horticultural programs has been associated with improvements for stress-related mental illness and burnout (Adevi & Lieberg, 2012; Sahlin, Ahlborg, Tenenbaum, & Grahn, 2015). A systematic review of gardening as a mental health intervention found overall reduced symptoms of anxiety and depression for a clinical population (Clatworthy, Hinds, & Camic, 2013). Additionally, therapeutic applications of green space have been found to reduce symptoms of clinical depression (Berman et al., 2012; Gonzalez, Hartig, Patil, Martinsen, & Kirkevold, 2010). Other reviews have found that nature walks were associated with a reduction in symptoms of anxiety and depression for clinical and nonclinical populations (Kotera, Lyons, Vione, & Norton, 2021) and, as an intervention for anxiety and depression, resulted in mental health improvements (Grassini, 2022). Access to activities within green spaces have also been found to reduce stress in psychiatric inpatient populations (Vujcic et al., 2017) and have the potential to reduce mental health admissions (Wheater et al., 2007).

However, there is a lack of synthesis of research exploring the effects of green space for people with diagnoses of severe mental health conditions, such as schizophrenia spectrum disorders (SSDs) (Tran et al., 2022). Therefore, the aim of this review is to identify and synthesize the evidence of the association of green space and mental health outcomes for people with SSDs. Green space interventions are promising due to their relatively low cost and accessibility (Bowen & Lynch, 2017; Bowen & Parry, 2015), with the potential to incur cost savings for the NHS (Wheater et al., 2007). Any identified benefits of green space could provide a rationale for preventative strategies, alongside integrating aspects of green spaces into therapeutic interventions and mental health services for this population. This review will include quantitative studies that explore the relationship between green space and SSDs and will address the following research questions:

1. What is the association between exposure to green space and the incidence of SSDs?
2. What are the benefits of exposure to green space for individuals with SSDs in relation to: (a) health service use, and (b) mental health symptoms?

## Method

This systematic review was pre-registered on PROSPERO (available at https://www.crd.york.ac.uk/PROSPERO/, ID: CRD42023431954) and conducted in accordance with the Preferred Reporting Items for Systematic Reviews and Meta-Analyses (PRISMA; Page et al., 2021). Databases were initially searched in July 2023, and an updated search was conducted in November 2023 (with no new papers identified). A PRISMA checklist is included as supplementary material.

### Inclusion and exclusion criteria

Inclusion criteria were: (1) articles of any date, published in English, with findings available; (2) involving exposure to green spaces, with green spaces defined as areas of vegetation (e.g. trees, grass, shrubs), adjacent to or within urban and rural areas, such as parks, gardens, forests, and nature reserves; (3) sample population of people with SSDs; (4) participants of any age (children to older adults); (5) quantitative studies (i.e. cross-sectional, cohort, experimental, correlational, longitudinal) reporting on either the relationship between exposure to green space(s) and SSDs or the benefits of green spaces for SSDs in relation to health service use and/or mental health symptoms; (6) outcomes of interest included reported risk of SSDs, health service use e.g., admission rates, and symptoms of SSDs and other related mental health outcomes e.g., anxiety, depression, etc.

Exclusion criteria were: (1) qualitative studies; (2) studies that did not include exposure to green space; (3) dissertations or theses; (4) existing reviews.

### Search strategy and sources of information

Seven electronic databases were searched, including PubMed (including MEDLINE), Web of Science, PsycARTICLES, APA, PsycINFO, CINAHL, ProQuest, and gray literature sources (EThOS, PsyArXiv, Open Science Framework). The following search terms were adopted: Psychosis OR psychoses OR psychotic OR schiz* OR paranoi* OR delusion* OR hallucinat* AND ‘Green space*’ OR ‘nature contact’ OR ‘urban nature’ OR ‘urban green’ OR ‘nature exposure’ OR ‘nature-based’ OR ‘nature experience’ OR ‘nature sound*’ OR ‘green area*’ OR greenspace* OR ‘natural space*’ OR ‘nature view*’

### Screening process

**Figure 1.**
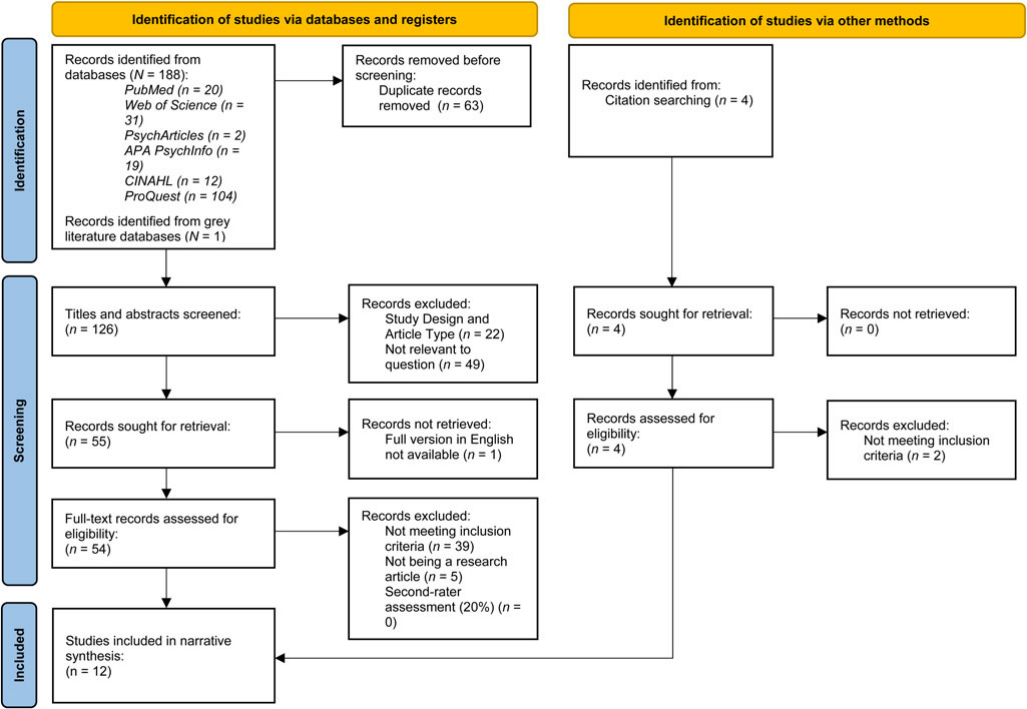
PRISMA flowchart.

Articles were initially identified by screening the title, abstract and subject or keywords, followed by full text screening. A second independent rater assessed 20% of all papers identified for full text screening using the outlined eligibility criteria. The search strategy and screening process are shown in Fig. 1.

### Quality assessment

The included studies were assessed for methodological quality using The Quality Assessment Tool for Quantitative Studies (Effective Public Health Practice Project; EPHPP, 2023). The EPHPP tool provides an overall rating of study methodology using the categories: ‘strong’, ‘moderate’ or ‘weak’, based on individual ratings for eight categories: study design, analysis, withdrawals and dropouts, data collection, selection bias, invention integrity, blinding as part of controlled trials, and confounders. Studies with two or more individual weak ratings are rated as weak overall. Studies with no weak ratings are rated as strong overall. This tool was used due to its ability to assess articles with a variety of quantitative study designs within the public health domain (Thomas, Ciliska, Dobbins, & Micucci, 2004). All of the included studies were rated independently by the first author and an independent rater, and there were no discrepancies in overall study quality ratings.

### Data extraction and synthesis

The main characteristics of each study and the study population were extracted, alongside data pertaining to the two research questions. A narrative synthesis approach was used, due to heterogeneity in study design, measurement of green space and reported outcomes. Only data relating to SSDs and green spaces were extracted and included in the analysis. Studies were grouped for synthesis according to the research questions they addressed.

## Results

The titles and abstracts of 126 records were screened; 54 records were extracted for full-text evaluation (including one paper from gray literature). Ten studies were eligible for inclusion. An additional two papers were found via searching the reference lists of eligible papers. Therefore, twelve papers were included in the final review (see Table 1 for a summary of study characteristics).

**Table 1.** Summary of studies and quality analysis

Author (Year)	Country	Study design	Total sample size (schizophrenia)	Green space measure or intervention	Outcome(s) of interest	Covariates measured	Quality assessment	Findings (related to schizophrenia spectrum disorders)
Bielinis et al. (2020)	Poland	Quasi-experimental	50 (23)	1 h 45 min of forest recreation intervention (walking, stretching, watching, landscapes).	Profile of Mood States (POMS). State-Trait Anxiety Inventory (STAI-S only).	None described.	Moderate	POMS: A significant decrease in tension-anxiety (*d* = 1.30), depression-dejection (*d* = 1.70), confusion (*d* = 2.01) and anger-hostility (*d* = 1.10) post intervention. A significant increase in vigor (*d* = 2.46) post intervention. No change in fatigue. STAI-S: A significant decrease in anxiety levels (*d* = 2.42) post intervention.
Boers et al. (2018)	The Netherlands	Cross-sectional	623 (623)	Percentage of agricultural, forest and natural areas (using Dutch land use database) within a circular buffer of 300 m around patient's home address.	Length of hospital admission (days).	Gender, age, urbanicity, socioeconomic status (individual).	Weak	Green space was not significantly correlated with length of hospital admission (Model 1: *t* = 0.232, *p* = 0.817; Model 2: *t* = 0.321, *p* = 0.748).
Chang et al. (2019)	Taiwan	Cohort study	869 484 (5069)	Normalized difference vegetation index (NDVI). *Higher values indicate greater healthy green vegetation.*	Schizophrenia incidence.	Gender, age, meteorological, health insurance rate.	Moderate	A significant negative association between surrounding greenness and schizophrenia risk (*p* < 0.05), with HRs reducing as NDVI increased from the 75th percentile onwards (Hazard ratio [HR] = 0.49, 95% confidence interval [CI] = 0.37–0.65; HR = 0.41, 95% CI = 0.30–0.56; HR = 0.41, 95% CI = 0.30–0.55; HR = 0.37, 95% CI = 0.25–0.55). The protective effects of green space were significant for both cities (HR = 0.22, 95% CI = 0.06–0.81) and metropolitan areas (HR = 0.46, 95% CI = 0.25–0.85).
Chang et al. (2020)	Taiwan	Cohort study	1 918 501 (3823)	NDVI and Enhanced vegetation index (EVI). Green space categories: Forest Recreational Descriptors of green spaces: Mean patch area Contiguity index Aggregation index	Schizophrenia incidence.	Gender, age, urbanicity, socioeconomic status (individual), meteorological, air pollution, health insurance rate.	Strong	Overall greenness associated with lower HRs for schizophrenia incidence (NDVI: HR = 0.79, 95% CI = 0.49–1.27; EVI: HR = 0.57, 95% CI = 0.27–1.19). A larger mean patch area^a^, higher contiguity index^b^, and higher aggregation index^c^ were associated with HRs < 1 for schizophrenia incidence, except for mean patch area in recreational green spaces (HR = 1.01, 95% CI = 0.97–1.05).
Engemann et al. (2018)	Denmark	Cohort study	943 027 (7609)	mean green space, spatial heterogeneity of green space. NDVI at 30 m^2^ resolution from Landsat archive.	Schizophrenia incidence.	Gender, age, socioeconomic status (individual).	Strong	Living at the lowest amount of green space was associated with a 1.52 (95% CI = 1.36–1.69, *p* < 0.000) fold increased risk of developing schizophrenia (IRR), compared to living at the highest level of green space. There was a dose-response relationship for exposure to green space at age 10 and risk of schizophrenia, with risk reducing as exposure increased. This association remained after adjusting for known risk factors for schizophrenia (urbanization, socioeconomic status, sex).
Engemann et al. (2019)	Denmark	Cohort study	943 027 (16 832)	NDVI at 30 m resolution from Landsat archive.	Incidence of psychiatric disorders (including schizophrenia, schizoaffective disorders, and schizophrenia and related disorders).	Urbanicity, parental age, socioeconomic status (parental), mental health history (parental), socioeconomic status (neighborhood).	Strong	Incident rate ratio (IRR) was higher for lowest NDVI compared to highest levels of NDVI for schizophrenia and schizophrenia and related disorders, but not for schizoaffective disorder (IRR = 1.33; 95% CI = 0.98–1.82). There was a dose-response relationship between IRR and NDVI for schizophrenia and schizophrenia and related disorders, with risk declining as NDVI levels increased.
Engemann et al. (2020a)	Denmark	Cohort study	943 027 (7609)	Two indicators: (1) land cover (Coordination of Information on the Environment; CORINE) and (2) vegetation density (NDVI)	Schizophrenia incidence.	Gender, age, socioeconomic status (individual), mental health history (parental), socioeconomic status (neighborhood).	Strong	HRs for schizophrenia showed decreased rates for children growing up in environments with more natural features, compared to children growing up in urban environments (agriculture HR = 0.69, 95% CI = 0.66–0.71; near-natural green space HR = 0.74, 95% CI = 0.63–0.88). There was a dose-response relationship for children growing up with near-natural green space as the most frequent land cover class.
Engemann et al. (2020b)	Denmark	Cohort study	19 746 (2636)	Mean yearly NDVI within square-shaped zones of 210 m × 210 (from birth to 10 years old)	Schizophrenia incidence.	Gender, age, socioeconomic status (individual), mental health history (parental).	Strong	Increasing NDVI was associated with decreased risk of schizophrenia (adjusted HR = 0.52, 95% CI = 0.40–0.66). Individuals in the highest NDVI exposure had a lower risk of developing schizophrenia (HR = 0.62, 95% CI 0.48–0.80), compared with individuals with the lowest NDVI.
Henson et al. (2020)	USA	Cohort study	63 (37)	GPS locations from smartphones matched to NDVI.	Ecological momentary assessment (EMA) survey which measured anxiety, depression, sleep, sociability, and psychotic symptoms.	Gender, age, socioeconomic status (individual), socioeconomic status (neighborhood), population density.	Moderate	Schizophrenia group: High NDVI settings were associated with significantly lower symptoms for anxiety (*d* = −0.70, *p* < 0.001), depression (*d* = −0.97, *p* < 0.001), and psychosis (*d* = −0.94, *p* < 0.001), compared to low NDVI settings. Schizophrenia group: High NDVI settings were associated with better sleep (*d* = −0.54, *p* < 0.001) but worse levels of sociability (*d* = 0.55, *p* < 0.001).
Kangarloo et al. (2023)	USA	Cohort study	35 (20)	Geo-locations from smartphones matched to NDVI.	EMA survey measuring emotional experiences using scales for happiness, sadness and anxiety. Affect expression in speech using Linguistic Inquiry and Word Count (LWIC).	Gender, age, education, employment status.		Small to moderate associations between greater greenspace exposure and lower average sadness (*ρ* = −0.05) and anxiety (*ρ* = −0.25) across the study duration. A moderate association between greater overall greenspace exposure and a lower proportion of negative affect words (*ρ* = −0.29). Specifically anxious (*ρ* = −0.26) and anger (*ρ* = −0.37) words. No significant daily associations between greenspace exposure and measures of anxiety or sadness, and negative affect words spoken.
Losert et al. (2012)	Germany	Cross-sectional	4198 (1586)	Percentage of forest area and percentage of agricultural area.	Admission rates.	Distance between town and psychiatric hospital.	Weak	An increase in surrounding agricultural land by 1% is related to a decrease in admissions by 4.3% (IRR = 0.96; *p* = 0.049). Findings for increases in forest area in relation to admission rates were not significant (IRR = 0.96. *p* = 0.076).
Rotenberg et al. (2022)	Canada	Cohort study	649, 020 (4841)	The Urban Health Equity Assessment and Response Tool (Urban HEART) measure of neighborhood-level green space.	Schizophrenia incidence.	Gender, age, socioeconomic status (neighborhood).	Strong	Neighborhoods with lowest amount of green space had a 24% higher risk of developing schizophrenia (adjusted IRR = 1.24, 95% CI = 1.06–1.45), compared to neighborhoods with the highest amount of green space.

a Size of greenspace area and edge.

b Connectedness of greenspaces within a location.

c Proximity to greenspace.

### Characteristics of included studies

The total number of participants with SSDs across all studies was 50 708. The studies were conducted in seven countries: Denmark (*k* = 4), Taiwan (*k* = 2), the USA (*k* = 2), Canada (*k* = 1), Germany (*k* = 1), the Netherlands (*k* = 1), and Poland (*k* = 1). Study designs included cohort (*k* = 9), cross-sectional (*k* = 2), and quasi-experimental (*k* = 1). Seven studies explored the incidence rates of SSDs in relation to green spaces, and five studies explored the effect of green spaces on individuals with SSDs in relation to mood (*k* = 3), anxiety (*k* = 3), symptoms of psychosis (*k* = 1), hospital admission rates (*k* = 1), and length of hospital admission (*k* = 1).

#### Sample characteristics

Four studies reported descriptives for gender for people with SSDs (Bielinis, Jaroszewska, Łukowski, & Takayama, 2020; Boers, Hagoort, Scheepers, & Helbich, 2018; Henson, Pearson, Keshavan, & Torous, 2020; Kangarloo et al., 2023), with a tendency towards male participants (range of 51–75%). Only two studies reported on participant ethnicity (Henson et al., 2020; Kangarloo et al., 2023), the samples were reported primarily as ‘White/Caucasian’ (35–54.3%). Four studies reported age descriptives, with ages ranging from 0–94 years (*μ_x̄_* = 42.87, ±13.82) (Bielinis et al., 2020; Boers et al., 2018; Henson et al., 2020; Kangarloo et al., 2023).

#### Measurement of green space

For studies exploring schizophrenia incidence, green space was quantified using five metrics: (1) normalized difference vegetation index (NDVI), a metric used to capture the presence and density of green vegetation over a patch of land (Chang, Wu, Pan, Lung, & Su, 2019; Chang et al., 2020; Engemann et al., 2018, 2019, 2020a, 2020b). NDVI calculations range from −1 to 1, where a value of 1 indicates the highest density of green cover (The National Aeronautics and Space Administration [NASA], 2000); (2) enhanced vegetation index (EVI), a more sensitive measure of green space, in which calculations range from 0 to 1, where 1 indicates the greatest density of healthy green vegetation (Chang et al., 2020); (3) categories of green space (e.g. forest and recreational green spaces) and descriptors of green space (e.g. area size, connectedness of spaces) (Chang et al., 2020; Engemann et al., 2018); (4) land cover from the Coordination of Information on the Environment (CORINE; European Environmental Agency, 2023), a database which classifies land cover according to categories ranging from urban green spaces to dense urban/industrial land use (Engemann et al., 2020a); and (5) The Urban Health Equity Assessment and Response Tool (Urban HEART; Centre for Research in Inner City Health, 2024), which provides a measure of neighborhood-level green space, calculating the average amount of green space per km^2^ in a circular buffer around residential areas, based on geospatial data (Rotenberg, Tuck, Anderson, & McKenzie, 2022).

For studies exploring the benefits of exposure to green spaces for SSDs in relation to health service use and/or mental health symptoms, one study measured green space exposure as a forest recreation intervention (walking, stretching, watching landscapes) (Bielinis et al., 2020). Two studies measured the percentage of agricultural, forest and natural areas within a circular buffer of patients' home addresses, using land databases (Boers et al., 2018; Losert, Schmauß, Becker, & Kilian, 2012). Two studies matched GPS locations from participants' mobile phones to NDVI data (Henson et al., 2020; Kangarloo et al., 2023).

#### Measurement of schizophrenia and mental health symptoms

SSDs were quantified using the following: (1) the International Classification of Diseases (ICD-8, ICD-9, ICD-10; WHO, 1968, 1993) (Bielinis et al., 2020; Chang et al., 2019, 2020; Engemann et al., 2018, 2019, 2020a, 2020b; Losert et al., 2012; Rotenberg et al., 2022); and (2) the Diagnostic and Statistical Manual of Mental Disorders (DSM-IV, DSM-V; American Psychiatric Association, 1994, 2001) (Boers et al., 2018; Henson et al., 2020; Kangarloo et al., 2023).

Other symptoms measured to assess the mental health benefits of green spaces included: (1) the Profile of Mood States (POMS; Dudek & Koniarek, 1987) (Bielinis et al., 2020); (2) the State-Trait Anxiety Inventory, state anxiety measure only (STAI-S; Spielberger, Gorsuch, Lushene, Vagg, & Jacobs, 1983) (Bielinis et al., 2020); (3) ecological momentary assessment (EMA; Shiffman, Stone, & Hufford, 2008), containing symptom questionnaires relating to anxiety and depression (Henson et al., 2020; Kangarloo et al., 2023), as well as symptoms of psychosis (Henson et al., 2020); (4) Linguistic Inquiry and Word Count (LWIC) for affect expression (Pennebaker, Booth, Boyd, & Francis, 2015) (Kangarloo et al., 2023).

#### Measurement of health service use

Health service use was quantified by: (1) length of hospital admission in days (Boers et al., 2018); and (2) psychiatric hospital admission rates, calculated by the number of admissions per location and analyzed as incidence rate ratios (IRRs). Only the first admission for each patient was counted and patients were excluded if their place of residence was unclear (Losert et al., 2012).

#### Quality analysis

After quality assessment, six of the included studies were rated as ‘strong’ (Chang et al., 2020; Engemann et al., 2018, 2019, 2020a, 2020b; Rotenberg et al., 2022), four were rated as ‘moderate’ (Bielinis et al., 2020; Chang et al., 2019; Henson et al., 2020; Kangarloo et al., 2023), and two were rated as ‘weak’ (Boers et al., 2018; Losert et al., 2012).

### Main findings

A summary of the studies included in the review is provided in Table 1.

#### What is the association between exposure to green space and the incidence of schizophrenia spectrum disorders?

Seven studies, all with a cohort design, explored the incidence rate of SSDs in relation to exposure to green spaces. Four studies took a developmental approach, focusing on childhood exposure to green spaces and risk of later development of SSDs (Engemann et al., 2018, 2019, 2020a, 2020b), whilst the remainder focused on adult exposure to green spaces. After quality analysis, six studies were rated as ‘strong’ and one was rated as ‘moderate’. Four studies calculated risk of SSDs using hazard ratios (HRs), where a HR < 1 indicates beneficial effects of green space exposure for reducing the risk of SSDs. Three studies calculated relative risk of SSDs using IRRs, to measure differences between low and high greenspace exposure. IRRs were calculated from measuring differing levels of green space exposure and associated incidence rates for SSDs, with higher IRRs indicating a greater risk of SSDs. IRRs > 1 indicated an increased risk from exposure, IRRs equal to 1 indicated no difference, and IRRS < 1 indicated beneficial effects of green space exposure.

*Exposure to green space is associated with a reduced risk of schizophrenia.* Studies reported reductions in schizophrenia risk for individuals with greater green space exposure, with HRs ranging from 0.62 for the lowest green space exposure (Engemann et al., 2020b), to 0.37 for the greatest greenspace exposure (Chang et al., 2019). Living in areas with the lowest concentration of green space was associated with an increased risk of developing schizophrenia (IRRs = 1.52 and 1.24), compared to living within the highest concentration of green space (Engemann et al., 2018; Rotenberg et al., 2022). One study found that overall neighborhood greenness, such as forests and recreational green spaces, was associated with lower HRs for schizophrenia incidence (NDVI HR = 0.79, EVI HR = 0.57) (Chang et al., 2020). HRs were found to be lower for children who had grown up in environments with near-natural features (i.e. vegetation ranging from grasslands to forests, containing human influences, such as benches and pathways), compared to those growing up in environments with urban as the most frequent land cover category (HRs = 0.69–0.74) (Engemann et al., 2020a), and HRs were lower for greater exposure to green space (HR = 0.62), compared to those with the lowest exposure (Engemann et al., 2020b). Regarding specific psychiatric diagnoses, one study found that the reduced risk only applied to schizophrenia and schizophrenia-related disorders for greater green space exposure, this effect was not found for schizoaffective disorders (Engemann et al., 2019). In addition to these findings, one study reported potential protective effects of exposure to green spaces within cities (HR = 0.22) and metropolitan areas (HR = 0.46), with increased areas of green space within these locations associated with reduced HRs (Chang et al., 2019). Associations remained across all studies after controlling for a number of covariates for schizophrenia risk, including: gender, age, socioeconomic status (individual, parents and neighborhood-level), urbanicity, and family mental health history (see Table 1).

*There may be a dose-response relationship between exposure to green space and schizophrenia risk.* Three studies reported a dose-response relationship between exposure to green space and schizophrenia risk, with risk reducing as exposure to green space increased (Engemann et al., 2018, 2019, 2020a), IRRs ranged from 1.52 at the lowest green space exposure to 1.18 at the highest green space exposure (Engemann et al., 2018). Increasing green space density and cover was associated with a decreased risk of schizophrenia (HR = 0.62) (Engemann et al., 2020b), with larger green spaces and greater proximity to green space associated with HRs < 1 (Chang et al., 2020). Another study reported a significant negative association between surrounding greenness and schizophrenia risk, with HRs ranging from 0.49 to 0.37 as green space density increased (Chang et al., 2019).

#### What are the benefits of exposure to green space for individuals with SSDs in relation to (a) health service use and (b) mental health symptoms?

Five studies explored the benefits of exposure to green space for individuals with SSDs. Two studies used a cross-sectional design (Boers et al., 2018; Losert et al., 2012), two studies used a cohort design (Henson et al., 2020; Kangarloo et al., 2023), and one study used a quasi-experimental design (Bielinis et al., 2020). After quality analysis, three studies were rated as ‘moderate’ (Bielinis et al., 2020; Henson et al., 2020; Kangarloo et al., 2023) and two were rated as ‘weak’ (Boers et al., 2018; Losert et al., 2012).

*(a) Health service use.* The two cross-sectional studies explored proximity to green space in relation to: (1) length of hospital admission (Boers et al., 2018), and (2) percentage of forest and agricultural areas in relation to admission rates for schizophrenia, calculated using IRRs (Losert et al., 2012). Findings suggest that proximity to green space was not significantly correlated with length of hospital admission (Boers et al., 2018). However, one study found a significant relationship between increases in the proportion of surrounding agricultural land and decreases in admission rates for people with SSDs (IRR = 0.96, *p* = 0.049) (Losert et al., 2012).

*(b) Mental health symptoms.* One cohort study measured symptoms of anxiety, depression, sleep, sociability, and psychotic symptoms amongst people with SSDs over the course of three months, an EMA survey accessed via mobile phone (Henson et al., 2020). GPS locations were collected alongside completion of the EMA, to measure green space cover and density. The study reported significantly lower symptoms for anxiety (*d* = −0.70), depression (*d* = −0.97) and psychosis (*d* = −0.94), and better sleep (*d* = −0.54) for settings with high levels of green space, compared to settings with low levels of green space.

Another cohort study measured emotional experience (happiness, sadness, and anxiety) and positive and negative speech affect (including negative affect subcategories: anxiety, anger, and sadness) over the course of seven days using EMA surveys accessed via mobile phones (Kangarloo et al., 2023). Data were collected three times a day at set times (10:00–13:00, 14:00–17:00, 17:00–20:00), and geolocations were collected alongside EMA data to measure green space cover and density. Results suggested small to moderate associations (rho values between −0.22 and −0.32) between greater green space exposure and lower scores for sadness and anxiety, across the seven days. There was also a moderate association (*ρ* = −0.29) between greater overall green space exposure and lower proportions of negative affect words used across the week, such as anxiety (*ρ* = −0.26) and anger words (*ρ* = −0.37). However, these findings were not significant at the daily level.

Finally, in a quasi-experimental study, 23 participants with SSDs participated in a forest recreation intervention, consisting of a one hour and 45-min walk in nature, with stretching and watching landscapes. The study captured pre and post-intervention scores using the POMS and the State-Trait Anxiety Inventory (STAI-S only). Significant decreases in all mood states of the POMS (except for vigor, which increased, and fatigue, where there were no changes) were found following the forest recreation intervention. There was also a significant decrease in anxiety levels (STAI-S), post intervention with a large effect size (*d* = 2.42).

## Discussion

This review aimed to synthesize the findings from quantitative studies that explored exposure to green space, incidence rates of SSDs and benefits for people with SSDs in relation to health service use and mental health symptoms. Twelve studies were included in the review, of which seven explored associations between green space exposure and SSD incidence, and five explored the benefits of green space exposure for people with SSDs.

Overall, the findings suggest that exposure to green space is associated with a reduced risk of SSDs, with some evidence that there may be a dose-response relationship. Childhood exposure to green space may also reduce the risk of SSDs later on. The quality of evidence was mostly high for these studies, and sample sizes were large, suggesting that we can be relatively confident in these conclusions. This supports existing literature reporting an association between green space exposure and reduced risk of depression and anxiety disorders (Brown et al., 2018; Gascon et al., 2018; Min et al., 2017; Sarkar et al., 2018). This body of evidence, taken together with the findings from the current review, provides clear evidence of the benefits of green space exposure in terms of reducing the risk of mental health diagnoses.

The studies in this review used a range of methods to explore the benefits of green space exposure, including cohort, cross-sectional and quasi-experimental designs. They report a range of benefits from green space exposure for people with SSDs, including improved mood, and reduced symptoms of anxiety and psychosis, as well as reductions in hospital admission rates. A strength of this body of literature is that a range of assessment methods have been used (e.g. EMA) such that it is not constrained by the sole use of self-report. However, the overall quality of the evidence was weaker, and sample sizes were much smaller, suggesting that we should be appropriately cautious when interpreting these findings. Nevertheless, these findings support existing literature showing that exposure to green space reduces symptoms of anxiety and depression (Berman et al., 2012; Gonzalez et al., 2010; Kotera et al., 2021) and reduces length of psychiatric hospital admissions (Wheater et al., 2007). Despite the limitations in quality, these studies offer promising implications of the potential benefits of green space exposure for people with SSDs, which should be investigated further in future research to provide more robust evidence.

Collectively, the findings from the review provide support for the need to integrate and maintain green spaces as a public health intervention (Maas et al., 2006; Soga et al., 2021). Benefits reported from increasing surrounding green space suggests that planning should take into account the proportion of available green spaces within urban settings, with increases in green spaces having risk-reducing effects for SSDs. Given the reported risk-reducing effects of childhood exposure to green spaces from the current review, measures could include increasing access to green spaces for children, such as parks and recreational activities within green spaces. In addition, people with SSDs may benefit from access to green space interventions, such as horticulture programs, walks and other activities in nature, as demonstrated for other mental health conditions (Clatworthy et al., 2013; Grassini, 2022; Kotera et al., 2021).

### Limitations

Regarding the literature included in the review, the evidence for the benefits of green space exposure for SSDs in relation to health service use and symptom reduction is emerging, such that the conclusions from this review are limited by the small number of studies available and the weaker quality of evidence, and findings are yet to be replicated. In addition, only two studies within the review reported on ethnicity, where the samples were majority White, and studies exploring incidence rates were mostly conducted in Denmark. Therefore, these results may not be generalizable cross-culturally.

It is also important to consider limitations of the review process. Omission of search terms for the full range of SSD symptoms may have biased results in favor of positive symptoms. Future studies should include search terms which encompass all dimensions of SSDs, including negative symptoms. In addition, the search terms for green spaces could be expanded to include components of green space, such as parks, woodlands, gardens, etc., to potentially increase eligible studies. Finally, this review did not investigate possible causes of heterogeneity for study results or complete sensitivity analyses, therefore the review is not able to determine the robustness of results beyond quality assessment.

### Recommendations for future research

This review highlights the need to develop the evidence base for the benefits of green space exposure for individuals with SSDs. It is notable that only one published study to date has used an experimental design to examine *in vivo* green space exposure and the effects on a range of mental health symptoms. Larger scale studies are needed to assess the benefits of exposure to green space using both experimental and longitudinal designs, examining a broader range of outcomes that are not solely focused on symptom reduction, including wellbeing and recovery, and to determine the ‘dose’ of green space exposure that is needed to produce clinically-significant change. Future studies are needed to understand the components of green space that might be particularly beneficial, to identify the mechanisms through which green space interventions work, and finally to identify the factors that might act as moderators to determine who might benefit most from green space interventions. Additionally, examining cross-cultural differences in green space exposure and SSDs should be a research priority, as well as determining whether the findings linking green space exposure and incidence rates for SSDs are replicated in other countries.

## Conclusion

Exposure to green space within both childhood and adulthood has risk reducing effects for the occurrence of SSDs, with some evidence for a dose response relationship. There is emerging evidence for the potential therapeutic benefits of exposure to green space for symptom reduction in people with SSDs and reduced health service use. Future research is needed to identify the optimal therapeutic ‘dose’ of green space exposure, to identify mediators and moderators of green space interventions and examine any cross-cultural differences.

## Supporting information

Marcham and Ellett supplementary material Marcham and Ellett supplementary material

## Data Availability

The datasets used during the review are available from the corresponding author on request.

## Supplementary material

The supplementary material for this article can be found at https://doi.org/10.1017/S0033291724001533.
